# The changing landscape of cardiac rehabilitation and the power of personalized therapy

**DOI:** 10.3389/fcvm.2024.1393217

**Published:** 2024-04-18

**Authors:** Jomme Claes, Tatiana Kuznetsova, Nicholas Cauwenberghs, Véronique Cornelissen

**Affiliations:** ^1^ Group Rehabilitation in Internal Disorders, Department of Rehabilitation Sciences, KU Leuven, Leuven, Belgium; ^2^ Hypertension and Cardiovascular Epidemiology, Department of Cardiovascular Sciences, KU Leuven, Leuven, Belgium

**Keywords:** cardiovascular rehabilitation (CR), home-based cardiac rehabilitation, hybrid cardiac rehabilitation, machine learning, patient profiling

## Introduction

Cardiovascular diseases (CVD) substantially burden our societal health and healthcare system. With 2.2 million deaths in females and 1.9 million deaths in males, CVD remain the leading cause of death and disability within Europe (1). Furthermore, in 2019 there were 12.7 million new cases and 113 million people were living with CVD in Europe (1). Cardiac rehabilitation (CR) mitigates this growing epidemic and is a class 1A recommendation in all guidelines (2), based on evidence indicating decreased morbidity and mortality, an increased quality of life (QoL), better physical functioning and good cost-effectiveness (3–5). To date, depending on the intervention (e.g., percutaneous interventions vs. cardiac surgery), patients with CVD are recommended to participate in a CR program starting within the first 6 weeks after treatment (2). Those CR programs have traditionally been delivered as ambulatory supervised centre-based programs, with patients visiting the hospital 2–3 times per week (2). However, uptake rates are low across Europe, with less than 40% of eligible patients participating after an acute event (2). The main barriers patients report for non-participation in a supervised centre-based program are intrapersonal (e.g., physical condition, presence of multiple co-morbidities, other responsibilities), interpersonal (e.g., social obligations, lack of social support), environmental (e.g., weather, distance, facilities) or organizational (e.g., lack of time, financial limitations) (6). If we are to achieve uptake rates of CR well above 50% such as recommended by the European Association of Preventive Cardiology quality indicators (2) or the United Kingdom's National Health Service Long Term Plan (7), we need to find ways to overcome these barriers. Therefore, traditional supervised centre-based CR model should not be seen as a one-size-fits-all approach, and we should aim for more individualization in CR prescription. Besides personalizing the content of the CR program to the individual patient, we should rethink how we offer these CR programs to our patients.

## Expanding the CR menu

In a study by Scherrenberg et al. only 10% of patients participating in a CR program expressed the desire for a fully supervised CR program (8). This underscores that the current one-size-fits-all strategy might never result in an optimal uptake of CR in daily practice.

In search for new strategies to widen access to CR and to improve implementation of the CR guidelines, alternative delivery methods such as home-based CR programs with or without remote monitoring were developed and validated (9). Home-based programs which incorporate remote monitoring and feedback have been shown to be equally effective in improving exercise capacity and physical activity in patients with CVD, at least in the short-term (10–14). Home-based programs can overcome some of the personal and practical barriers related to centre-based CR (6). Yet, several important new challenges were identified including (1) the availability and adoption of technology, (2) a decrease in adherence over time, (3) a selection bias towards digitally literate patients, and (4) patients' need for in person contact with a clinical team (15). Additionally, the American Association of Cardiovascular and Pulmonary Rehabilitation suggests that home-based CR should be used in low-to-moderate risk patients as it is not yet clear if home-based CR can guarantee the same levels of exercise modalities, safety and efficacy in high risk populations (16). This could explain why according to the study of Scherrenberg et al. only 36% of patients believed in remote CR as a stand-alone solution (8).

Therefore, ongoing research focusses more on hybrid delivery models which combine the strengths of supervised and home-based CR. Hybrid CR can evoke a satisfactory exercise intensity and lead to similar short-term effects on exercise capacity and quality of life compared to supervised centre-based CR (17–19). This form of CR was deemed a legitimate option for 54% of CR participants in the study of Scherrenberg et al. (8). However, multiple definitions of hybrid CR currently exist. Some programs incorporate centre-based and home-based CR simultaneously, with centre-based sessions equally dispersed over time or diminishing throughout time, while others start up the home-based program after a period of supervised centre-based CR. In general, hybrid CR has shown to be equally effective as centre-based CR, but with higher adherence and lower costs (18). Long-term results about the adherence to an adequate physically active lifestyle after hybrid CR are scarce, but it could be hypothesized that a guided translation of exercise into the home environment could lead to better long-term results due to e.g., increased self-efficacy.

## Finding the right CR program for the right patient

If we put effort in expanding the CR menu, we should also ensure that patients find their way to the most individually suitable CR delivery method. Patient commitment to a certain CR delivery method depends on a plethora of personal, environmental and organizational factors. These should be taken into account when selecting the most fitting program and two possible strategies to achieve this goal are presented.

### Letting the patient pick

The most intuitive approach would be to let patients choose their preferred delivery method of CR and several recent studies have investigated this approach (20–25), but each with substantial limitations and shortcomings. First, the content of the CR programs differed substantially, with marked differences in exercise interventions (frequency, intensity, time and type) between the different delivery modes or even comparisons between exercise interventions and educational programs, making a direct comparison invalid (24, 26). Second, a cross-sectional observational study design was often used with the main outcomes being questionnaires aiming to investigate barriers and enablers to different CR delivery modes (21, 23). Third, no study has investigated the effect of providing a choice of delivery mode on uptake, adherence or health outcomes. It is possible that patients make a choice based on perceived “ease” and not because it is the logical choice given their circumstances. This could lead to a less effective treatment because exercise parameters (e.g., duration and intensity) are less likely to be respected. Although the patients' choice remains important to consider, we should not only look for the CR program the patient wants, but also for the program the patient needs.

### Considering the patient profile

A second approach to find the best match between patient and program consists of incorporating the patient profile as a determinant in the decision process. A large retrospective study by Tang et al. using the UK National Audit of Cardiac Rehabilitation (NACR) database, found that patients choosing home-based CR differed significantly from the populations in centre-based or hybrid CR and had lower completion rates of their CR program (26). Given that home-based programs were developed to increase uptake and adherence to CR in populations who are otherwise difficult to reach, these data suggest that the program did not reach the right patients. We recently applied machine learning (ML) approaches (Support Vector Machine and Random Forest) to predict adherence to a technology enabled home-based CR platform which could clearly distinguish adherence patterns based on baseline patient characteristics (12, 27). In a study by Gaalema et al. a Classification and Regression Tree (CART) was used to determine which patient characteristics were most important to predict the number of sessions completed in a supervised centre-based CR program. They found that current smoking, lower socio-economic status, younger age and non-surgical diagnosis associated with a lower adherence (28). Similarly, Pack et al. developed a multivariable logistic regression model to predict the drop-out risk in a centre-based CR program and identified the same type of patients who dropped out before completing 12 sessions (29). Interestingly, they also developed a simplified tool to easily predict the drop-out risk before enrolling the patient in supervised centre-based CR to allow for program adjustment or to suggest alternatives which might fit the patient's profile better and thus result in a higher adherence rate.

## Discussion and future perspectives

We aimed to raise awareness on the importance of increasing uptake and adherence of CR for the fast growing group of patients with CVD and highlight the urgent need for the validation of alternative CR delivery methods across the globe. Moreover, we wanted to draw attention on how ML can assist in matching patients to the most suited CR program based on their individual profiles.

Increasing uptake and adherence of CR will lead to better health outcomes in the long term and thus contribute to a decreased personal and societal burden of CVD (30). A recent study by Hinde et al. estimated that any 1% increase in uptake of CR can save £2,567,312 in the UK alone (7). In addition, using ML to profile patients and to develop tools for use by clinicians to support patients in their decision-making process could contribute to a better allocation of resources, saving expensive supervised centre-based CR spots for those patients that need them the most.

Offering personalized CR delivery methods and supporting patients towards the most likely suitable form of CR should become the standard care in hospitals. Rolling out these CR programs in a real world setting will allow for the gathering of real-world data, which not always corresponds with the results obtained in RCTs, as shown by Tang et al. (26). In approximately half of European countries ≥1 CR programs offer alternative CR delivery methods, but this does not mean that each program in the country does so and it still leaves many countries who offer only centre-based CR (31).

Finally, we want to highlight that the patients' needs might change over time. Instead of seeing CR as a fixed solution, we advocate to see CR as a continuum through which the patient can move flexibly in time. For optimal risk management, we should offer patients a menu of CR programs, regularly evaluate the chosen CR strategy and adjust it if needed. Future initiatives towards alternative delivery methods and tools to refer the patient to the most personalised program are therefore still encouraged.
